# *De novo* transcriptome sequencing and analysis of male, pseudo-male and female yellow perch, *Perca flavescens*

**DOI:** 10.1371/journal.pone.0171187

**Published:** 2017-02-03

**Authors:** Yan-He Li, Han-Ping Wang, Hong Yao, Paul O’Bryant, Dean Rapp, Liang Guo, Eman A. Waly

**Affiliations:** 1 Fish Genetics and Breeding Laboratory, The Ohio State University South Centers, Piketon, Ohio, United States of America; 2 College of Fisheries, Huazhong Agricultural University, Wuhan, Hubei, PRC; Leibniz Institute on aging - Fritz Lipmann Institute (FLI), GERMANY

## Abstract

Transcriptome sequencing could facilitate discovery of sex-biased genes, biological pathways and molecular markers, which could help clarify the molecular mechanism of sex determination and sexual dimorphism, and assist with selective breeding in aquaculture. Yellow perch has unique gonad system and sexual dimorphism and is an alternative model to study mechanism of sex determination, sexual dimorphism and sexual selection. In this study, we performed the *de novo* assembly of yellow perch gonads and muscle transcriptomes by high throughput Illumina sequencing. A total of 212,180 contigs were obtained, ranging from 127 to 64,876 bp, and N50 of 1,066 bp. The assembly RNA-Seq contigs (≥200bp) were then used for subsequent analyses, including annotation, pathway analysis, and microsatellites discovery. No female- and pseudo-male-biased genes were involved in any pathways while male-biased genes were involved in 29 pathways, and neuroactive ligand receptor interaction and enzyme of trypsin (enzyme code, EC: 3.4.21.4) was highly involved. Pyruvate kinase (enzyme code, EC: 2.7.1.40), which plays important roles in cell proliferation, was highly expressed in muscles. In addition, a total of 183,939 SNPs, 11,286 InDels and 41,479 microsatellites were identified. This study is the first report on transcriptome information in Percids, and provides rich resources for conducting further studies on understanding the molecular basis of sex determinations, sexual dimorphism, and sexual selection in fish, and for population studies and marker-assisted selection in Percids.

## Introduction

Yellow perch, *Perca flavescens*, natively distributes from central Canada, east and southeast through the Great Lakes—St. Lawrence and the upper Mississippi basins and on the Atlantic slope from Maine to Georgia [1]. It is a vital recreational and commercial species in Chesapeake Bay and the Great Lakes [2]. Yellow perch females outgrow males starting from 8–11 cm in total length and also have dominant and subordinated females [3]. Yellow perch have an XY sex-determination system [4]. Uniquely, yellow perch female have only one ovary and spawn egg ribbons (all the eggs in a ribbon-like bag). Also sex-reversed pseudo-males develop a single gonad [4]. Male skewed sex ratios have been observed in experimental and natural populations (Wang et al. unpublished data). Thus, yellow perch doubtlessly attracted much attention for its value as the material of studying mechanism of sexual dimorphism, sex determination, and sexual selection.

Gonad is the primary organ presenting morphological signs of sexual dimorphism, and its development is referred as sex differentiation [5]. Muscle could be regarded as a tissue presenting growth signs and would show the expression profile of growth related gene. Studies of sex differences in expression assume that genes that are expressed more in males encode male traits, and genes expressed more in females encode female traits, and this assumption is a key foundation to genetic studies of sexual dimorphism and sexual conflict. Sex-biased genes are the product of different male- and female-specific evolutionary pressures and therefore can be used to measure sex-specific selection with molecular data [6].

RNA-Sequencing has showed the advantages of identification of species-specific genes [7], revealing sexually dimorphic gene expression [8] and the availability of a large number of genetic markers developed is facilitating trait mapping and marker-assisted breeding [9]. More and more studies started to focus on the gonad transcriptome analysis and provided archives for future studies in molecular mechanisms of sexual dimorphism and evolution [10–12]. The patterns of tissue-associated gene sex-biased expression could provide more valuable resources for discovering the molecular mechanism of sexual dimorphism in size, ornaments, behavior, and traits [13].

However, there is no study identifying and characterizing the sex-related genes in yellow perch at the transcriptomic level. This study was intent to quantify and identify sex/tissue-related genes from gonads and muscles using RNA-seq and find what pathways they are involved in. The findings from this study may provide valuable transcriptomic resource for studying molecular mechanisms of sexual dimorphism and developing molecular markers in yellow perch. The findings also could assist the selection breeding of yellow perch and other fish species in aquaculture in general.

## Results

### Transcriptome sequencing and assembly

**Fig 1:**
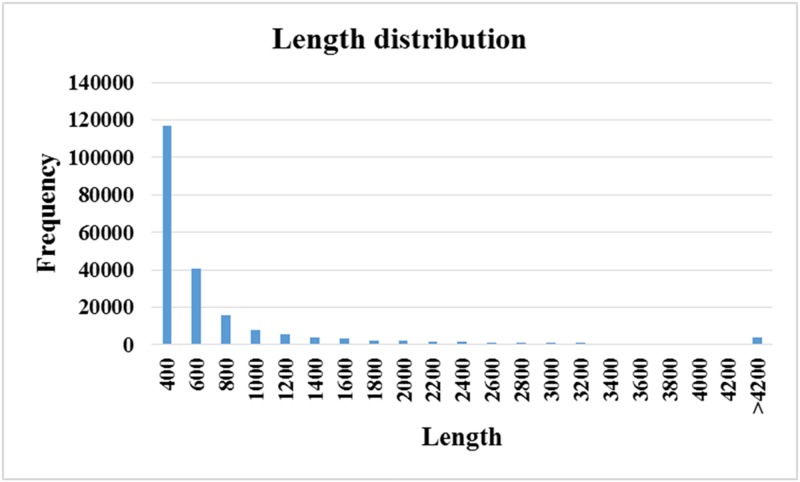
Length distribution of contigs assembled.

From Illumina sequencing results, a total of 572,074,124 paired-end reads were generated. The raw transcriptome sequences in the present study have been submitted to the National Center for Biotechnology Information (NCBI) Short Read Archive (SRA) site, the accession number is SRP074479. The low-quality sequences and ambiguous nucleotides were trimmed, and then the remaining high-quality reads, a total of 548,699,676 (95.9%), were obtained for transcriptome assembly and analysis (S1 Table). A total of 212,180 contigs were assembled, ranging from 127 to 64,876 bp, and the average length was 695 bp, with the N50 value of 1,066 bp (Table 1). The contigs less than 200 bp were removed and finally, a total of 211,976 contigs (length distribution see Fig 1) were for blasting and annotating, and 30.98% of all contigs were less than 301bp (≤ 300bp). The workflow of transcriptome data analysis was shown in S1 Fig.

**Table 1:** Statistics of yellow perch transcriptome sequences assembly.

**Summary statistics**	
Reads count	548,699,676
Matched reads count	486,557,128
Not matched reads count	62,142,548
Number of contigs	212,180
Reads in pairs	378,170,740
Broken paired reads	95,485,390
Average length of reads (bp)	97,94
**Contigs measurements (including scaffolded regions)**	
N75(bp)	419
N50(bp)	1,066
N25(bp)	2,916
Minimum length (bp)	127
Maximum length (bp)	64,876
Average length (bp)	695
Count	212,180
Total length	147,493,106
**Contigs measurements (excluding scaffolded regions)**	
N75(bp)	392
N50(bp)	908
N25(bp)	2,371
Minimum length (bp)	74
Maximum length (bp)	54,724
Average length (bp)	636
Count	231,356
Total length	147,146,312

### Mapping reads to contigs

The reference sequence is the assembly sequence containing 211,976 contigs which are not less than 200 bp. Of the clean reads from six libraries, for the NF gonad (NFG), 66.49% were reads mapped in pairs, 14.21% were reads not mapped; for NF muscle (NFM), 62.74% were reads mapped in pairs, 18.47% were reads not mapped; for NM gonads (NMG), 76.90% were reads mapped in pairs, 12.95% were reads not mapped; for NM muscle(NMM), 64.85% were reads mapped in pairs, 16.15% were reads not mapped; for PM gonads (PMG), 68.83%, 12.40% were reads not mapped; for PM muscle (PMM), 62.28% were reads mapped in pairs, 18.35% were reads not mapped (S2 Table).

### Functional annotation

**Fig 2:**
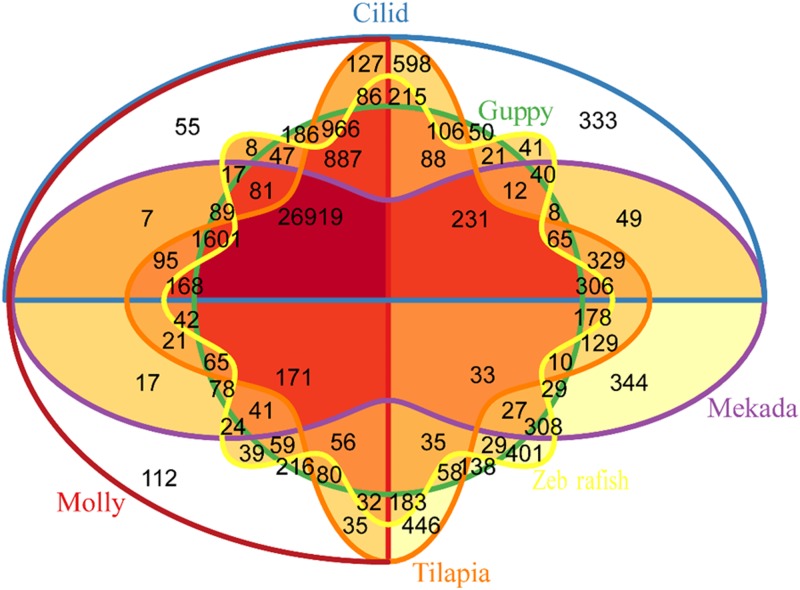
The Venn diagram of genes distributions in the six fish (zebrafish, tilapia, cichlid, guppy, medaka and amazon molly).

Of 211,976 assembled contig sequences, a total of 36,117 (17.04% of all contigs assembled) contigs had significant hit as unigenes after searching against the six reference protein sequences including Zebrafish (*Danio rerio*), Tilapia (*Oreochromis niloticus*), Cichlid (*Haplochromis burtoni*), Amazon molly (*Poecilia formosa*), Guppy (*Poecilia reticulata*), and Medaka (*Oryzias latipes*) by standalone blast+ programs [14]. Based on blasting similarity among them, there are 26,919 unigenes shared among six teleost fish species (Fig 2).

**Fig 3:**
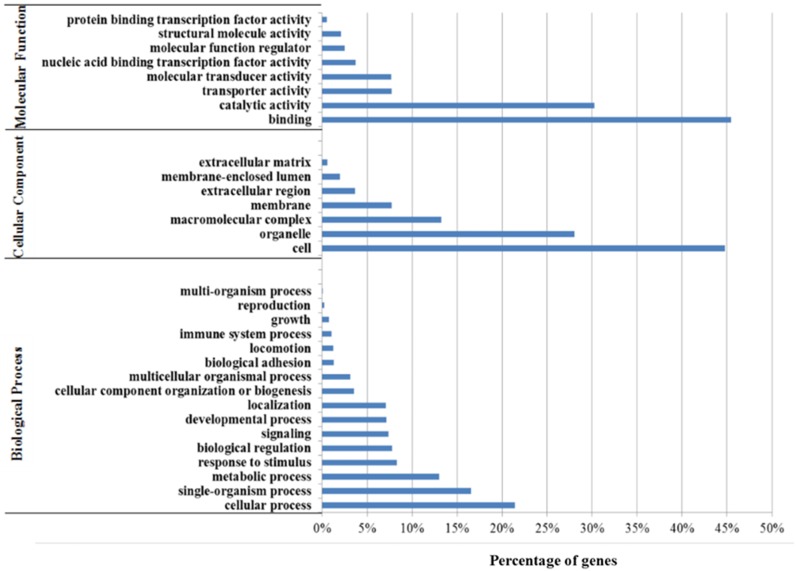
Functional classification of yellow perch unigenes based on three main Gene Ontology (GO) categories: biological process, molecular function and cellular component.

The unigenes were then used to perform gene ontology (GO) annotation by Blast2GO. With a total of 72,801 GO assignments, the GO analysis showed that 16,038 annotated unigenes were assigned at least one GO term. For the biological process, cellular process (GO: 0009987) was the most represented category, and genes involved in single-organism process (GO: 0044699), metabolic process (GO: 0008152), response to stimulus (GO: 0050896), biological regulation (GO: 0065007), localization (GO: 0051179), developmental process (GO: 0032502); and signaling (GO: 0023052) were also highly represented. For molecular function, binding (GO: 0005488) was the most represented category, followed by catalytic activity (GO: 0003284), transporter activity (GO: 0005212) and molecular transducer activity (GO: 0060089). For cellular component, cell (GO: 0005623) was the most represented category, followed by organelle (GO: 0043226), macromolecular complex (GO: 0032991) and membrane (GO: 0016020) (Fig 3). Of all GO categories, the biological process ontology was the most prevalent, followed by molecular functions ontology and cellular component ontology. Within biological process ontology, a total of 307 unigenes were identified related to growth (GO: 0040007), 2,969 unigenes were annotated to developmental process, 93 unigenes were annotated to reproduction.

The contigs assembled with previously identified gene matches were carried for functional pathways analysis based on the KEGG (Kyoto Encyclopedia of Genes and Genomes) database. The results showed that 14,277 unigenes were categorized into different functional groups (Table 2). The largest functional group was organismal system (29.13%, 4,159), including immune system (983), endocrine system (1,039) and nervous system (787). Unigenes grouped into metabolism (4,046, 28.34%), included global and overview maps (1,547), carbohydrate metabolism (479), lipid metabolism (439), amino acid metabolism (379) and glycan biosynthesis and metabolism (306). Environmental information on processing, cellular process groups, and genetic information processing contained 3,096 (21.69%), 1,689 (11.83%) and 1,287 (9.01%) unigenes, respectively.

**Table 2:** KEGG biomedical mapping for yellow perch.

KEGG categories represented		Number of unigene sequences	Number of mapped KO
Metabolism		4,046	2,984
	Global and overiew maps	1,547	1,239
	Carbohydrate metabolism	479	312
	Energy metabolism	195	130
	Lipid metabolism	439	281
	Nucleotide metabolism	240	174
	Amino acid metabolism	379	290
	Metabolism of other amino acids	109	67
	Glycan biosynthesis and metabolism	306	227
	Metabolism of cofactors and vitamins	165	130
	Metabolism of terpenoids and polyketides	36	32
	Biosynthesis of other secondary metabolites	46	32
	Xenobiotics biodegradation and metabolism	105	70
Genetic information processing		1,287	1,054
	Transcription	189	158
	Translation	441	355
	Folding, sorting and degradation	441	346
	Replication and repair	216	195
Enviromental information processing		3,096	2,030
	Membrane transport	28	28
	Signal transduction	2,635	1,679
	Signaling moleculars and interaction	433	323
Cellular processes		1,689	1,149
	Transport and catabolism	491	342
	Cell motility	191	118
	Cell growth and death	422	325
	Cellular community	585	364
Organismal systems		4,159	2,594
	Immune system	983	630
	Endocrine system	1,039	651
	Circulatory system	271	163
	Digestive system	400	244
	Excretory system	158	88
	Nervous system	787	499
	Sensory system	140	82
	Development	240	149
	Enviromental adaptation	141	88
Total		14,277	9,811

### Sex and tissue specifically expressed genes and involved pathways

Based on the transcriptome mapping data, 93, 1,440 and 3 contigs were identified as specifically expressed in pseudo-male, male and female respectively; 9,476 and 858 contigs were identified as specifically expressed in gonads and muscles (S3 Table). However, only 4 (1), 55 (29) and 0 (0) contigs were blasted to match genes (mapped to GO terms) for pseudo-male, male and female respectively (S4 Table); 696(396) and 45 (25) contigs were blasted to match genes (mapped to GO terms) for gonads and muscles (some of them listed in S5 Table). Of sex specifically expressed ORFs searching, 19 ORFs were found specifically expressed in male, 6 ORFs specifically expressed in pseudo-male, and no ORF specifically expressed in female (S3 Table; S2 Fig).

**Fig 4:**
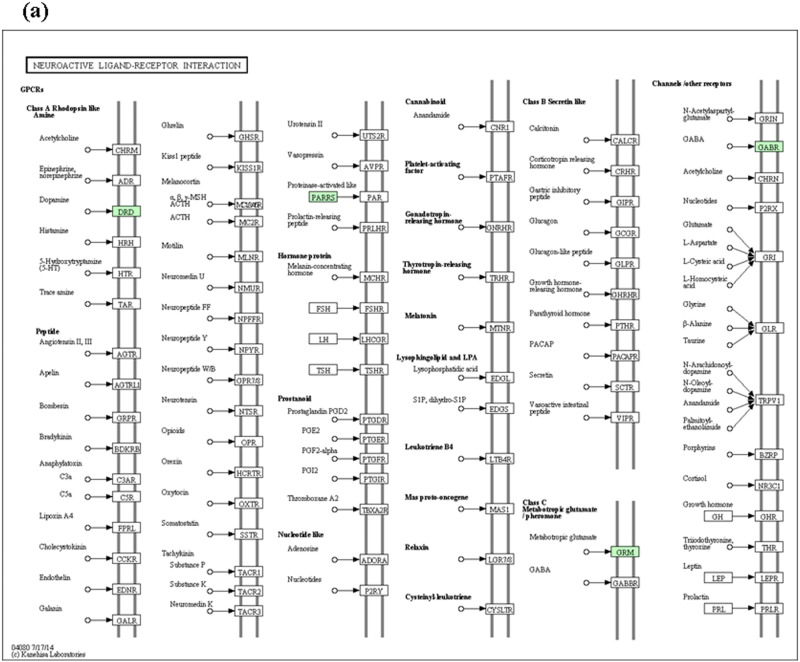
Neuroactive ligand-receptor interaction KEGG map for male yellow perch. The genes identified being involved in pathways were shown in green boxes.

**Fig 5:**
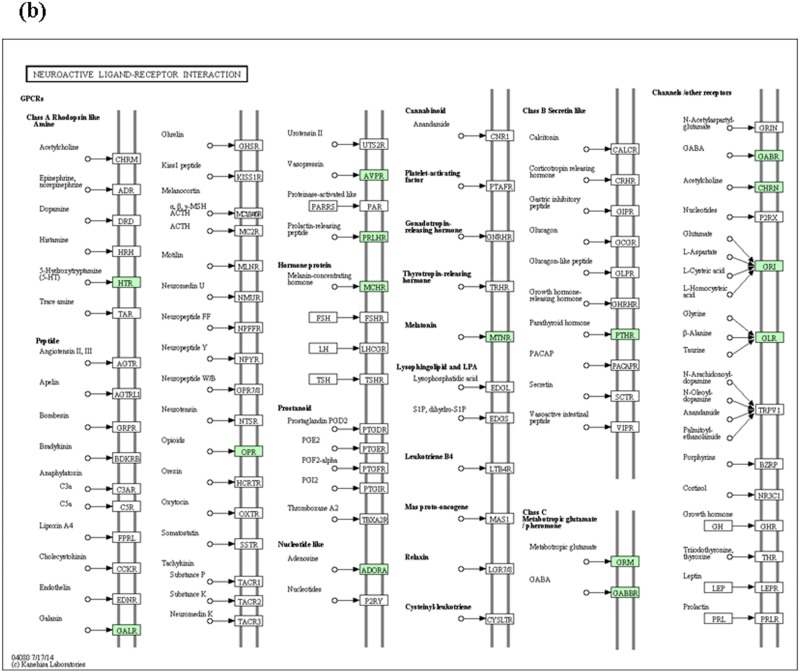
Neuroactive ligand-receptor interaction KEGG map for gonads of yellow perch. The genes identified being involved in pathways were shown in green boxes.

The contigs identified respectively as specifically expressed in pseudo-male, male and female were further carried for functional pathway analysis to identify biological pathway that shows sexual dimorphism in yellow perch based on the KEGG database. The number of sex-biased genes was counted in different pathway categories (S6 Table). However, there is no pseudo-male- and female-biased gene involved in any pathway. Male-biased genes were involved in 29 pathways, and the Enzyme and related pathways involved for genes specifically expressed in male showed in S6 Table. Of the functional pathways, trypsin (enzyme code, EC: 3.4.21.4) and neuroactive ligand-receptor interaction (Fig 4) were most specifically expressed in yellow perch males. Similarly, the pathway analysis for tissue specifically expressed genes showed that an enzyme named pyruvate kinase (enzyme code, EC: 2.7.1.40) was involved in most pathways for muscle specifically expressed genes, and two pathways involving most genes for gonad specifically expressed genes were neuroactive ligand-receptor interaction (Fig 5) and metabolic pathways (S7 Table). Besides, for gonad specifically expressed genes, several pathways associated with gonadal development and sex maintenance were found, including Oocyte meiosis, GnRH signaling pathway, TGF-beta signaling pathway, Oxytocin signaling pathway, and Ovarian steroidogenesis. Additionally, TNF signaling pathway and Apoptosis pathway were found in gonads. Of the enzymes involved, c-Jun N-terminal kinase (enzyme code, EC: 2.7.11.24) and TGF-beta receptor type-1 (enzyme code, EC: 2.7.11.30) were most expressed in gonads (S7 Table).

### SNPs, InDels and microsatellites discovery

A total of 183,939 SNPs and 11,286 InDels were identified. These SNPs and InDels were located on 39,362 contigs, in a total length of 71,759,949 bp and the estimated frequency of SNPs was 2.59 per kb. The number of substitution for A/G, C/T, A/T, A/C, G/T and C/G was 52,498, 56,834, 17,553, 20,460, 21,907 and 14,687, respectively and the transition/transversion (Ts/Tv) ratio was about 1.46. Among these SNPs, 111,727 were located in ORF and 25,172 were missense mutation. A total of 41,479 microsatellites were identified from 18,210 unigene sequences (Table 3). The most frequently repeated motifs were dinucleotide repeats (accounted for 77.14% of all microsatellites identified), followed by tri- (17.42%), tetra- (5.03%), penta- (0.27%), and hexa-nucleotide repeats (0.14%). Based on female, male, and pseudo-male specifically expressed contig sequences, no microsatellite loci, 133 and four repeat motifs were identified from them respectively. Based on tissue specifically expressed contigs, 93 and 1,036 repeat motifs were identified in muscles and gonads respectively.

**Table 3:** Statistics of microsatellites identification in yellow perch.

Microsatellites identified	
Transcriptome sequences	41,479
Di-nucleotide repeats	31,996
Tri-nucleotide repeats	7,226
Tetra-nucleotide repeats	2,088
Penta-nucleotide repeats	112
Hexa-nucleotide repeats	57
Number of microsatellites containing sequences	18,210
NF specifically expressed contigs	0
any nucleotide repeats	0
NM specifically expressed contigs	133
Di-nucleotide repeats	110
Tri-nucleotide repeats	15
Tetra-nucleotide repeats	8
Number of microsatellites containing sequences	110
PM specifically expressed contigs	4
Di-nucleotide repeats	4
Number of microsatellites containing sequences	4
Number of microsatellites with sufficient flanking sequence (≥ 50 bp) for PCR primer design	
Muscles specifically expressed contigs	93
Di-nucleotide repeats	76
Tri-nucleotide repeats	12
Tetra-nucleotide repeats	3
Penta-nucleotide repeats	1
Hexa-nucleotide repeats	1
Number of microsatellites containing sequences	74
Gonads specifically expressed contigs	1,036
Di-nucleotide repeats	824
Tri-nucleotide repeats	159
Tetra-nucleotide repeats	50
Penta-nucleotide repeats	3
Number of microsatellites containing sequences	847

Forty-eight primer pairs were randomly selected for primer synthesis and validation, and 27 primer pairs were successful in PCR amplification using genomic DNA from 12 individuals of *P*. *flavescens*. The remaining 21primer pairs failed to generate PCR products in one or some individuals. Of 27 primer pairs, 7 primer pairs generated multiple bands that included the target bands and a larger band (>1,000bp). The rest of 20 primer pairs only amplified the target bands. The eight microsatellite loci selected from 48 microsatellite loci, were used to analyze the polymorphism assessment in one hatchery population of *P*. *flavescens*. Seven microsatellite loci are polymorphic. The number of alleles varied from 0 to 14, and observed heterozygosity ranged between 0.205 (YPM6) and 0.89 (YPM46) (Table 4).

**Table 4:** The primer sequences and their polymorphism assessment results.

Locus name	Primer sequences (5′−3′)	Ta(°C)	Observed no. of alleles	Size range(bp)	N	HObs	HExp	PIC
YPM 6	CAGTCGGGCGTCATCAAAGCTGATAGGCGTGCTGAT	55	4	187–208	32	0.156	0.205	0.195
	ACTTTAAAACACAGGGACAGGTCT							
YPM27	CAGTCGGGCGTCATCAATTGGGCCTGCAGTTATTTT	55	8	108–205	25	0.92	0.876	0.842
	TGTGAGTGTGTGAGTGTGAGTGT							
YPM41	CAGTCGGGCGTCATCACCCACCTGCAGAACACTACA	55	4	210–231	31	0.387	0.342	0.32
	GTTTAACCCCACGAAAATGC							
YPM30	CAGTCGGGCGTCATCAGACACGGAAATGGAAAAAGGT	55	11	174–210	31	1	0.705	0.645
	CCAGCTGGAGAAACAGGACT							
YPM20	CAGTCGGGCGTCATCAGGACCACTCGTTTTGCTCTG	55	6	246–276	31	0.548	0.499	0.464
	CACAAACACATGCACACTCG							
YPM34	CAGTCGGGCGTCATCAGTTATCATGCTGGGGAGGAC	55	2	190–206	32	0.406	0.329	0.271
	CAAACCTCATCACAGGAGCA							
YPM46	CAGTCGGGCGTCATCAGCTAAAAGAAACTCCTGGCAAA	55	14	173–218	28	0.929	0.89	0.861
	GCGGATCAAACGAAAGAAAA							
YPM17	CAGTCGGGCGTCATCACCTGCCTCCTGAACCTTTCT	55	na	na	na	na	na	na
	TGCTACGACACTGCCTGCTA							

## Discussion

### Transcriptome sequencing, assembly and annotation

Transcriptome sequencing using Illumina platforms technology provides resources for gene expression profiling studies as well as identification of molecular markers, alternative splice variants and RNA editing events [15]. For example, through Illumina platforms, sex differentially expressed genes were identified in *S*. *marmoratus* [10], sex differentiation and potential sex determining genes were identified in *P*. *margaritifera* [11], a nearly complete source of transcriptomic sequence as well as marker information for sharpsnout seabream (*Diplodus puntazzo*) [12]. These studies focused on sex differentially expressed genes and markers information. The present study focused on applications of Illumina platforms sequencing to transcriptome analysis of yellow perch and laid the foundation for further sex dimorphism and determination research. The transcriptomes of gonads and muscles from female, male and pseudo-male were sequenced on Illumina platforms and annotated to different biological process ontology and functional pathways.

In the present study, 17.04% of all contigs assembled (36,117) had a significant hit as unigenes, which was lower than that obtained from flounder (22.31%) and sturgeon (22.05%), and similar to crucian carp (17.44%) [7,16,17]. The low percentage of matched sequences might be due to 1) short contigs occupying large percentage, however, the significance of a BLAST comparison depends in part on the length of query sequences [18]; 2) the parameters for blasting setting the significance higher, and only getting the highest significant similar sequences; 3) blast method. In this study, 30.98% of the unigene sequences were not very long (≤ 300 bp). The parameters for blasting are: e-value cutoff 1e-10, best hit score edge 0.05, best hit overhang 0.25 and max target seqs 1. The standalone blast+ programs were used to blast the query transcriptome sequences against the six reference protein sequences including Zebrafish, Tilapia, Cichlid, Amazon molly, Guppy, and Medaka. The possibility that yellow perch may have more unique genes compared to other species was not excluded. However, the absolute number of contigs matched from yellow perch (36,117) was higher than flounder (21,697), sturgeon (12,189) and crucian carp (22,273) [7, 16, 17]. Thus, the contigs from trascriptome sequencing and blasting were good enough for further annotation.

### Sex specifically expressed genes and ORFs

Four and 55 genes were identified for pseudo-male and male respectively, and the genes identified were studied further for pathway analysis. Sex specifically expressed ORFs (Open Reading Frames) were searched, and 19 ORFs were found specifically expressed in males, 6 ORFs were specifically expressed in pseudo-males. As the literature we searched, there are no references about the potential function of the sex specifically expressed ORFs, and whether the ORFs locate on the sex chromosomes of yellow perch is not known so far. Further experiments would be cloning these genes and investigating their functions since these genes are not reported presently but expressed specifically. However, there was no female-biased gene/ORF identified in this study.

About the sample FG, The detected value of RNA RIN was always low in the experiment. However, we found the FG didn't degrade even though the value of RIN is low. When the oocyte becomes competent to undergo fertilization, the oocyte itself produce some molecules including maternal mRNAs, RNAs, proteins, carbohydrates, lipids, hormones and vitamins, which are important for the proper development of the embryo [19]. The 5S rRNA is one important molecule involved considerably in this process. For example, up to 75% of the total RNA from mullet ovaries was 5S rRNA [20]. As we know, the RIN value is lower if the 5S rRNA band is brighter. The cDNA library was constructed based on capturing the mRNA molecules. Thus, this might be the reason of that the number of reads in FG is lower than other samples in this study. The female-specific expressed contigs might more exist in the 5S rRNA bands in the stage of yellow perch we collected. This might be the reason of lacking female-specific contigs in this study. The findings could provide the reference for collecting samples in the future for investigating the sex-specific expressed genes in ovary.

In previous study, females were significantly larger than males starting from at least one-year-old fish (data not published), however, unfortunately, in this study we didn’t find any specifically expressed contigs in female. The specifically expressed contigs in muscle are showed in S5 Table. However, we didn’t find the annotated genes specifically related to growth expressed in muscle, such as *Igf*, *Igfr* or *Myob*.

### Pathway analysis of sex and tissue specifically expressed genes

Of the functional pathways involved in normal males, pathway of neuroactive ligand receptor interaction had the highest hits, and enzyme of trypsin (enzyme code, EC: 3.4.21.4) was highly expressed. The neuroactive ligand-receptor interaction and metabolic pathways were involved in most gonad specifically expressed genes. The neuroactive ligand-receptor interaction, calcium signaling pathways, cell cycle and DNA replication pathways were testified to be associated with lactation performance in mice, and genes associated with neuroactive ligand-receptor interaction and calcium signaling pathways were significantly upregulated and positively correlated with lactation performance [21]. As we know, fish have no lactation performance as mammals do. However, we believed that these pathways to some extent might be correlated with gonad development of fish. Generally, most one-year-old yellow perch males could reach sexual maturity although some documents reported that age for yellow perch at maturity was generally two years [22]. In the present study, the yellow perch we sampled were slightly older than one year old. The neuroactive ligand-receptor interaction was only found in males and gonads, respectively, which maybe to some extent indicated that the pathway was correlated with sexual maturity and the genes involved in the pathway might regulate the gonad development. Additionally, TNF signaling pathway and apoptosis pathway was only found in males, which to some extent was attributed to the sampled males being sexually mature and the testes sampled were in the degenerating stage. The regular spawning season for yellow perch is generally from March to April [23]. Samples were collected in the middle of May. Further experiments will be carried out to testify these hypotheses and inferences.

For pathway analysis of muscle specifically expressed genes, more transcripts were found for an enzyme named pyruvate kinase (enzyme code, EC: 2.7.1.40) being involved in most pathways. Pyruvate kinase is a key glycolytic enzyme, and is known to be the rate-limiting step of glycolysis [24]. The role of pyruvate kinase is complex and it plays a part in the nucleus not only as a pro-proliferative, but also pro-apoptotic stimuli [25]. Generally, pyruvate kinase plays important roles in cell proliferation that only proceeds when metabolism is capable of providing adequate metabolic intermediates to ensure both energy regeneration and the synthesis of cell building blocks in sufficient amounts [26, 27]. Pyruvate kinase determines whether glucose is converted to lactate for regeneration of energy or used for the synthesis of cell building blocks [28]. Presently, pyruvate kinase M2 has been extensively studied in cancer research, and it was found that RNA interfering (RNAi) targeting PKM2 significantly inhibited tumor growth [24, 29, 30]. However, to our knowledge, there is no any document that reported pyruvate kinase research in aquaculture animals. Then what role(s) pyruvate kinase play(s) in the growth of yellow perch and what the gene expression profiles are would be interesting for aquaculture researchers. In the present study, pyruvate kinase was only found in muscles instead of gonads, which could, in part, indicate that the fish sampled were still in the stage of vigorous growth while the gonads of the fish were not in the stage of growing development.

No female- and pseudo-male-biased genes were involved in any pathways while male-biased genes were involved in 29 pathways. The results might indicate that: 1) female and pseudo-male yellow perch had much the same genomic resource including sex-related genes; 2) female and pseudo-male were in much the same special stage and had much the same trascriptomic profile; 3) there exists but cannot be identified based on the present databases. Yellow perch have sexual size dimorphism that females were significantly larger than males starting from at least one-year-old fish [31], since lower growth rate was observed in mature males [32, 33], and estimates of female age and length at 50% maturity ranged from 1.0 to 5.8 years and from 15.7 to 20.1 cm, respectively, while male age and length at 50% maturity ranged from 1.2 to 2.1 years and from 9.4 to 12 cm [34]. To some extent, the results from pathway analysis also showed that the sexual size dimorphism among female, male, and pseudo-male did not yet occur significantly in yellow perch when sampling was performed. However, males and females have presented different transcriptome profiles. Besides, based on specifically expressed genes and ORFs identified, the results from pseudo-males presented some difference as those from females, which to some extent indicated that the transcriptome profile of pseudo-male started to present a differentiation from female. Further experiments would be needed to investigate the correlations between sexual size dimorphism and transcriptome profiles including the pathway analysis and gene expression profiles.

Several sexual dimorphic biological pathways were found for gonad specifically expressed genes including oocyte meiosis, GnRH signaling pathway, TGF-beta signaling pathway, oxytocin signaling pathway, and ovarian steroidogenesis. The c-Jun N-terminal kinase (enzyme code, EC: 2.7.11.24) and TGF-beta receptor type-1 (enzyme code, EC: 2.7.11.30) were most expressed in gonads. However, no sex-biased gene was involved in sexual dimorphic biological pathways. That means, of sexual dimorphic biological pathways, there is no difference for female, male, and pseudo-male of the sampled yellow perch. Nevertheless, the differential expression profile of the genes involved in sexual dimorphic biological pathways may be different and needed to be further investigated.

### Transcriptome-derived SNPs, InDels and microsatellites discovery

Molecular genetic markers are essential to study genetic diversity, population genetic structure, evolution, and ecology [35, 36]. Next generation sequencing provides a good resource for the development of molecular genetic markers because of the enormous amount of sequence data that it generates [37]. Transcriptome sequencing is a next generation sequencing based on mRNA [38]. The RNA-seq sequences can provide an excellent source for mining and development of molecular markers. Molecular genetic markers based on transcriptome sequences are not only useful for studying genetic diversity and population genetic structure, but also for the detection of functional variation and gene associated genetic analysis. Marker-assisted selection has great potential to increase production and identify the sex in aquaculture molecular breeding.

In the present study, a total of 183,939 SNPs and 11,286 InDels were identified. The estimated frequency of SNPs was far lower than 4.28 per kb in *Larimichthys crocea* population [39] and 3.58 per kb in *Larimichthys crocea* individual [40]. Such low frequency of SNPs might be because: 1) the nine fish were descended from a single mate-pair and the frequency of SNP in the study more reflected the frequency in individual than population; 2) only the reads properly located on the same contigs were extracted to call SNPs, which would make these contigs more reliable and also threw away a small part of true mutations; 3) the model used in SNP calling was based on diploid, which would filter out relatively low frequency mutations, and the SNP calling procedures were expected to improve the true positive frequency.

Based on 211,976 assembled contigs, 18,210 unigene sequences were identified to contain total 41,479 microsatellites. About 8.6% of the transcriptomic sequences possess microsatellites. This microsatellite frequency was a little higher than *Macrobrachium nipponense* (8.2%), but slightly lower than *Carassius auratus*(8.9%)[16, 41]. The microsatellite frequency based on transcriptomic sequences sometime depends on the search parameters for mining microsatellites, such as the repeat length threshold and the number of repeat motifs unit. In this study, the mononucleotide repeat motifs were excluded, which is consistent with the studies of *Macrobrachium nipponense* and *Carassius auratus*. The software used to identify microsatellites loci also can affect the microsatellite frequency. Some tools can detect imperfect microsatellites (e.g., Sputnik) while others detect perfect microsatellites (e.g., MISA and Mreps). This study and the other studies in *Macrobrachium nipponense* and *Carassius auratus* used the tools that can detect perfect microsatellites (MISA for this study and that of *Carassius auratus*; Mreps software for the study of *Macrobrachium nipponense*) to discover microsatellites. The di-nucleotide repeats (accounted for 77.14% of all microsatellites identified) were the most frequent repeats, followed by tri- (17.42%), tetra- (5.03%), penta- (0.27%), and hexa-nucleotide repeats (0.14%). The results were consistent with the microsatellite distributions based on RNA-seq reported [16, 39]. In this study, microsatellites were also identified from female, male, pseudo-male muscles, gonads, and tissue specifically expressed contigs sequences. However, as we know, it is impossible to find tissue specifically owned microsatellite loci since all the cells in the body have the same DNA [42]. Similarly, it is hard or even impossible to find sex specifically owned microsatellite loci since female and male share nearly identical genomes except the sex chromosome [43]. But here, the analyses could provide some reference for finding and cloning specifically expressed genes easier since the specifically expressed contigs were correlated with specific genes. Through designing primers based on the repeat motifs and PCR amplifications, maybe new specifically expressed gene(s) or marker(s) would be found and cloned. To tentatively validate the microsatellites identified, 48 primer pairs were randomly selected for primer synthesis, and 27 primer pairs were found to be amplified successfully in all individuals tested. Of the eight microsatellite loci selected from 48 microsatellite loci, seven one are polymorphic. The results showed that the microsatellites identified from transcriptome sequencing data could be developed for yellow perch to find useful polymorphic microsatellites. In the next study, we will collect more yellow perch populations to further identify more polymorphic microsatellites from the microsatellites identified.

## Conclusions

A total of 211,976 contigs were obtained and 183,939 SNPs, 11,286 InDels and 41,479 microsatellites were identified from assembled contigs. No female- and pseudo-male-biased genes were found being involved in any pathways. Male-biased genes were found being involved in 29 pathways, of which neuroactive ligand receptor interaction was highly involved. Pyruvate kinase was highly expressed in muscle. This study is the first report on transcriptome information in Percids, and provides rich resources for conducting further studies on understanding the molecular basis of sex determinations, sexual dimorphism and sexual selection in fish, and for population studies and marker-assisted selection in Percids. Further experiments would be needed to test the roles of the enzyme pathways involved and the gene expression profiles associated with those pathways.

## Materials and methods

### Ethics statement

All animal care and experimental procedures were approved by the Institutional Animal Care and Use Committee of Ohio State University.

### Sample preparation and collection

A pair-mating was conducted in the breeding season of 2013 at the Aquaculture Research Center, the Ohio State University South Centers to generate a full-sib family. The experimental fish were fed twice a day and reared at 24±2°C in 400 L round tanks in the green house. One-half of four-week old fry from the family were subjected to a testosterone treatment to produce a sex-reversed male or all-male population. When the fish reached ~10 gram, the reversed fish were PIT tagged and then combined with another half of mixed sex population fish and raised in a single 2-meter round tank. In 2014, three normal males (NM), three normal females (NF) and three pseudo-males (PM) were sampled from the tanks when they were 1+ year-old. Males with a single gonad were identified as pseudo-males because yellow perch females have only one gonad. Gonads and muscles were dissected from those fish which were anaesthetized by MS-222 and stored in RNAlater^®^ stabilization solution (Life technologies, USA) over 24 hours at– 4°C, then stored at– 80°C until total RNA isolation.

### RNA isolation and cDNA library construction

Total RNA was isolated (gonads and muscles) using TRIzol^™^ (Invitrogen, Carlsbad, CA) according to the manufacturer’s protocols. The concentration was quantified using NanoDrop spectrophotometry (NanoDrop Technologies, Wilmington, DE, USA), and the quality of total RNA was checked by electrophoresis in 1% agarose gel and Agilent 2100 Bioanalyzer (Agilent Technologies, Palo Alto, CA, USA). The RNA with good integrity was utilized to construct the library. The cDNA library was constructed using a Truseq^®^ Stranded mRNA Sample Prep kit (Illumina, USA) according to the manufacturer’s protocols. Finally, the six cDNA libraries were sequenced on an Illumina platform using paired-end strategy.

### Illumina sequencing and *de novo* assembly

Sequence reads (2*100 bp pair-end sequencing) pools were generated on an Illumina platform from the Ohio State University Molecular and Cellular Imaging Center. The raw data were deposited in the NCBI SRA site and can be found under accession number SRP074479. Quality filtering of sequence data was performed using FastQC. Before using the RNA-seq reads for de novo assembly, a Create Sequencing QC Report was run using CLC Genomics Workbench v7.5.1 (following called CLC; QIAGEN Aarhus A/S; www.clcbio.com) to produce the quality reports. Low-quality sequences and the adaptors were trimmed using Trim Sequences tools in CLC, and then the trimmed sequences were checked by running the Create Sequencing QC Report again. The clean reads of all six samples were assembled to one sequence as referenced by *de no* assembly tool in CLC. The parameters for the *de novo* assembly were set as: the Word size was set to 20, the Automatic bubble size was set to 50, the Minimum contig length was set as default value, the option of “Perform scaffolding” was not checked. The optimal assembly results were chosen according to an evaluation of the assembly encompassing the total number of contigs, the distribution of contig lengths, the N50 statistic and the average coverage. The assembled transcripts were based on the main isoform of each transcript, and only contigs with lengths of greater than 200 bp were included in the downstream analysis. Mapping each sample RNA-seq reads to the reference sequence using the RNA-Seq Analysis tool implemented in CLC. The parameters for mapping reads back to the contigs were: the option of Map reads back to contigs (slow) were selected (Mismatch cost, 2; Insertion cost, 3; Deletion cost, 3; Length fraction, 0,5; Similarity fraction, 0,8) and the option of Update contigs were chosen.

### Annotation

In this study, we checked the redundant contigs using CD-HIT program (http://www.bioinformatics.org/cd-hit/) while the assembly contigs (≥200bp) were using for annotation, and just found about 4.7% (211,836/211,976) is redundant. Thus, we decided to analyze the assembly results without removing redundant contigs instead of restarting the annotation. The assembly RNA-Seq contigs (≥200bp) (S1 File) were used for a similarity search program against reference protein sequences including zebrafish (*Danio rerio*), tilapia (*Oreochromis niloticus*), cichlid (*Haplochromis burtoni*), amazon molly (*Poecilia formosa*), guppy (*Poecilia reticulata*), and medaka (*Oryzias latipes*). The similarity searches were performed using the BLASTX program [14] with the parameters of e-value cutoff 1e-10, best hit score edge 0.05, best hit overhang 0.25 and max target seqs 1. The combined blast results were used to Gene ontology (GO) annotation analysis using Blast2GO [44]. The level two GO terms associated with transcriptome contigs were retrieved and then the annotation results were categorized as biological process, molecular function, and cellular components. KEGG (Kyoto encyclopedia of Genes and Genomes) pathways were assigned to those assembled contigs using online KEGG Automatic Annotation Server (www.genome.jp/tools/kaas) [45].

### Sequence data mapping

The trimming reads from six samples were mapped to the reference sequence assembled from the de novo assembly above, respectively. Sequence data mapping was performed using the tool of RNA-Seq Analysis in CLC. CLC Reference Mapper was run with default settings (mismatch cost = 2, insertion cost = 3, deletion cost = 3, length fraction = 0.8 and similarity fraction = 0.8).

### Identification of expressed genes related to sex and tissue

The reads per kilobase of exon per million mapped reads (RPKM) values were calculated from the mapping performance by the tool of RNA-Seq Analysis in CLC. Then the mapping reads were used to construct different groups of comparison using the Set Up Experiment in CLC. The Kal’s test was used to identify the differentially expressed genes with p-value <0.05 based on the transformed data with log2. ORFs were searched using TransDecoder (http://transdecoder.github.io/).

### SNPs, InDels and microsatellite discovery

The pair-end reads above from each sample were merged together (S1 File) and SNPs and InDels were called using diploid model. The reads were mapped using BWA-MEM version 0.7.13 [46]. The alignment file was sorted and marked duplication using SortSam and MarDuplicatesWithMateCigar programs from the software package (http://broadinstittute.github.io/picard). The RealignerTargetCreator and IndelRealigner programs from the GATK version 3.5 [47] was used to realign the alignments around InDels. The UnifiedGenotyper function in GATK was used to call SNPs and InDels with read mapping quality at least 60 and base quality at least 30. The SNPs and InDels with a depth higher than 20, a quality score greater than 60 and without significantly different (*P* = 0.95) mapping quality, base quality and read position between reference and alternative base were treated as putative mutations.

The assembly unigene dataset (S1 File) and the sex-specific expressed unigene datasets were used for detecting microsatellite loci by a Perl script known as MicroSAtellite (MISA, http://pgrc.ipk-gatersleben.de/misa) [48]. Six types of microsatellites were identified with criteria of di- to hexa-nucleotides motifs, and the minimum repeat unit was defined as 6 for di-, 5 repeats for tri-, tetra, penta-, and hexa-nucleotides. The sequences composed of two or more repeat units with motifs separated by >100 bp were considered to be two or more microsatellites. And the sequences detected as microsatellites were collected for future primer designing if the microsatellite sequences have flanking sequences of ≥50 bp on both sides.

The 48 microsatellite primers were designed by the Primer3 (http://biotools.umassmed.edu/bioapps/primer3_www.cgi) [49] and using the parameters as following: (1) PCR product size ranging from 100 to 300; (2) primer length range of 18 to 27 bases; (3) primer Tm of 53–63°C, with 60°C as the optimum Tm; (4) GC content between 20% and 80%, with an optimum of 50%. The primers were synthesized by Eurofins U.S. Company (http://www.eurofins.com). PCR was performed in 10 μL reaction volumes containing 5 μL JumpStart^™^ REDTaq^®^ ReadyMix^™^ Reaction Mix (SIGMA), 0.5 μL of each primer (10 μM) and 0.5 μL DNA (100 ng/μL) as template. A polymerase chain reaction was performed as following: 95°C for 5 min, followed by 35 cycles of denaturation at 95°C for 30 sec, 57°C for 45 sec, and elongation at 72°C for 45 sec, followed by a 10 min extension at 72°C. The PCR products were detected by 1.2% gel agarose electrophoresis. The eight primers selected from 48 primers were synthesized with labelled by FAM, ROX or HEX for further analyzing polymorphism assessment in one hatchery population of *P*. *flavescens* (n = 32). Polymerase chain reaction of 6 uL contained 1.5 pmoL of both nontailed and labelled primers, and 0.1 pmoL of the tailed primer, 3 uL of JumpStart RedMix (Sigma), 25 ng DNA, in the presence of 100μM spermidine. Amplification was conducted in PTC-200 thermal cyclers (MJ Research) using an initial denaturation at 95°C for 3 min, followed by 35 cycles of 30 s denaturation at 95°, 30 s annealing at a locus-specific temperature (Table 4), 30 s extension at 72°, and a final 7-min extension at 72°. Amplification products DNA fragment analysis using ABI 3130 genetic analyzer.

## Supporting information

S1 Table Number of raw reads, gene counts and specifically expressed genes of yellow perch transcriptome sequencing samples. (DOCX)

S2 Table Mapping results of six tissue samples. (DOCX)

S3 Table Overview of sex-biased genes/ORFs identification. (DOCX)

S4 Table Sex specifically expressed genes identified in male and pseudo-male. (XLSX)

S5 Table Tissue specifically expressed genes identified. (XLSX)

S6 Table The Enzymes and related pathways involved in female, pseudo-male and male of yellow perch. (DOCX)

S7 Table The Enzymes and related pathways involved in muscles and gonads in yellow perch. (XLSX)

S1 Fig The workflow of transcriptome data analysis. (DOCX)

S2 Fig The ORFs specifically found in female, male and pseudo-male. (DOCX)

S1 File The transcriptome dataset for SNPs running. (ZIP)

S2 File Assembly sequences obatained. (ZIP)

## References

[1] Grzybowski M, Sepulveda-Villet OJ, Stepien CA, Rosauer D, Binkowski F, et al: Genetic variation of 17 wild perch populations from the Midwest and East Coast analyzed via microsatellites. *Trans Am Fish Soc* 2010, 139:270–287.

[2] Blazer VS, Pinkney AE, Jenkins JA, Iwanowicz LR, Minkkinen S, et al: Reproductive health of yellow perch Perca flavescens in selected tributaries of the Chesapeake Bay. *The Science of the total environment* 2013, 447:198–209. 10.1016/j.scitotenv.2012.12.088 23384644

[3] Lynn SG: Cloning and expression of key endocrine genes in a study on estrogen stimulated sexual size dimorphism (SSD) in yellow perch (Doctoral Dissertations). University of Kentucky 2006.

[4] Malison JA, Kayes TB, Best CD, Amundson CH, Wentworth BC: Sexual Differentiation and Use of Hormones to Control Sex in Yellow Perch (Perca flavescens). *Can J Fish Aquat Sci* 1986, 43:26–35.

[5] Lu J, Luan P, Zhang X, Xue S, Peng L, et al: Gonadal transcriptomic analysis of yellow catfish (Pelteobagrus fulvidraco): identification of sex-related genes and genetic markers. *Physiological genomics* 2014, 46(21):798–807. 10.1152/physiolgenomics.00088.2014 25185028

[6] Marin I, Baker BS: The Evolutionary Dynamics of sex determination. *Science* 1998, 281(5385):1990–1994. 9748152 10.1126/science.281.5385.1990

[7] Fan Z, You F, Wang L, Weng S, Wu Z, et al: Gonadal transcriptome analysis of male and female olive flounder (Paralichthys olivaceus). *BioMed research international* 2014, 2014:291067 10.1155/2014/291067 25121093 PMC4121151

[8] Ayers KL, Davidson NM, Demiyah D, Roeszler KN, Grutzner F, et al: RNA sequencing reveals sexually dimorphic gene expression before gonadal differentiation in chicken and allows comprehensive annotation of the W-chromosome. *Genome biology* 2013, 14(3):R26 10.1186/gb-2013-14-3-r26 23531366 PMC4053838

[9] Cardoso-Silva CB, Costa EA, Mancini MC, Balsalobre TW, Canesin LE, et al: De novo assembly and transcriptome analysis of contrasting sugarcane varieties. *PloS one* 2014, 9(2):e88462 10.1371/journal.pone.0088462 24523899 PMC3921171

[10] Sun L, Wang C, Huang L, Wu M, Zuo Z: Transcriptome Analysis of Male and Female Sebastiscus marmoratus. *PLoS ONE* 2012, 7(11):e50676 10.1371/journal.pone.0050676 23209808 PMC3507777

[11] Teaniniuraitemoana V, Huvet A, Levy P, Klopp C, Lhuillier E, et al: Gonad transcriptome analysis of pearl oyster Pinctada margaritifera: identification of potential sex differentiation and sex determining genes. *BMC Genomics* 2014, 15:491 http://www.biomedcentral.com/1471-2164/1415/1491. 10.1186/1471-2164-15-491 24942841 PMC4082630

[12] Manousaki T, Tsakogiannis A, Lagnel J, Sarropoulou E, Xiang JZ, et al: The sex-specific transcriptome of the hermaphrodite sparid sharpsnout seabream(Diplodus puntazzo). *BMC Genomics* 2014, 15:655 http://www.biomedcentral.com/1471-2164/1415/1655. 10.1186/1471-2164-15-655 25099474 PMC4133083

[13] Sharma E, Künstner A, Fraser BA, Zipprich G, Kottler VA, et al: Transcriptome assemblies for studying sex-biased gene expression in the guppy, Poecilia reticulata. *BMC Genomics* 2014, 15:400 http://www.biomedcentral.com/1471-2164/1415/1400. 10.1186/1471-2164-15-400 24886435 PMC4059875

[14] Camacho C, Coulouris G, Avagyan V, Ma N, Papadopoulos J, et al: BLAST+: architecture and applications. *BMC Bioinformatics* 2009, 10:421 10.1186/1471-2105-10-421 20003500 PMC2803857

[15] Ozsolak F, Milos PM: RNA sequencing: advances, challenges and opportunities. *Nature reviews Genetics* 2011, 12(2):87–98. 10.1038/nrg2934 21191423 PMC3031867

[16] Liao X, Cheng L, Xu P, Lu G, Wachholtz M, et al: Transcriptome Analysis of Crucian Carp (Carassius auratus), an important aquaculture and hypoxia-tolerant species. *PloS one* 2013, 8(4):e62308 10.1371/journal.pone.0062308 23630630 PMC3632525

[17] Vidotto M, Grapputo A, Boscari E, Barbisan F, Coppe A, et al: Transcriptome sequencing and de novo annotation of the critically endangered Adriatic sturgeon. *BMC Genomics* 2013, 14:407 http://www.biomedcentral.com/1471-2164/1414/1407. 10.1186/1471-2164-14-407 23773438 PMC3691660

[18] De Wit P, Pespeni M, Ladner J, Barshis D, Seneca F, et al: The simple fool's guide to population genomics via RNA-Seq: an introduction to high-throughput sequencing data analysis. *Ecology Resources* 2012, 12:1058–1067. 10.1111/1755-0998.12003 22931062

[19] Lubzens E, Young G, Bobe J, Cerdaá J: Oogenesis in teleosts: how fish eggs are formed. *General and Comparative Endocrinology* 2010, 16: 367–389. 10.1016/j.ygcen.2009.05.022 19505465

[20] de Cerio OD, Rojo-Bartolomeé I, Bizarro C, Ortiz-Zarragoitia M, Cancio I: 5S rRNA and Accompanying Proteins in Gonads: Powerful Markers Identify Sex and Reproductive Endocrine Disruption in Fish. *Environmental Science & Technology* 2012, 46: 7763–7771. 22724546 10.1021/es301132b

[21] Wei J, Ramanathan P, Martin IC, Moran C, Taylor RM, Williamson P: Identification of gene sets and pathways associated with lactation performance in mice. *Physiological genomics* 2013, 45(5):171–181. 10.1152/physiolgenomics.00139.2011 23284081

[22] Munro CL, Macmillan JL: Growth and overpopulation of yellow perch and the apparent effect of increased competition on brook trout in long lake, halifax county, Nova scotia. *Proceedings of the Nova Scotian Institute of Science* 2012, 47(1):131–141.

[23] Kristan J, Stejskal V, Policar T: Comparison of Reproduction Characteristics and Broodstock Mortality in Farmed and Wild Eurasian Perch (Perca fluviatilis L.) Females During Spawning Season Under Controlled Conditions. *Turkish Journal of Fisheries and Aquatic Sciences* 2012, 12(2):191–197.

[24] Guo W, Zhang Y, Chen T, Wang Y, Xue J, et al: Efficacy of RNAi targeting of pyruvate kinase M2 combined with cisplatin in a lung cancer model. *J Cancer Res Clin Oncol* 2011, 137(1):65–72. 10.1007/s00432-010-0860-5 20336315 PMC11827957

[25] Mazurek S: Pyruvate kinase type M2: a key regulator of the metabolic budget system in tumor cells. *Int J Biochem Cell Biol* 2011, 43(7):969–980. 10.1016/j.biocel.2010.02.005 20156581

[26] Ye J, Mancuso A, Tong X, Ward PS, Fand J, et al: Pyruvate kinase M2 promotes de novo serine synthesis to sustain mTORC1 activity and cell proliferation. *PNAS* 2012, 109(18):6904–6909. 10.1073/pnas.1204176109 22509023 PMC3345000

[27] Lunt SY, Muralidhar V, Hosios AM, Israelsen WJ, Gui DY, Newhouse L, et al.: Pyruvate kinase isoform expression alters nucleotide synthesis to impact cell proliferation. *Mol Cell* 2015, 57(1):95–107. 10.1016/j.molcel.2014.10.027 25482511 PMC4289430

[28] Gruning NM, Rinnerthaler M, Bluemlein K, Mulleder M, Wamelink MM, et al: Pyruvate kinase triggers a metabolic feedback loop that controls redox metabolism in respiring cells. *Cell Metab* 2011, 14(3):415–427. 10.1016/j.cmet.2011.06.017 21907146 PMC3202625

[29] Mazurek S, Boschek CB, Hugo F, Eigenbrodt E: Pyruvate kinase type M2 and its role in tumor growth and spreading. *Semin Cancer Biol* 2005, 15(4):300–308. 10.1016/j.semcancer.2005.04.009 15908230

[30] Christofk HR, Vander Heiden MG, Harris MH, Ramanathan A, Gerszten RE, et al: The M2 splice isoform of pyruvate kinase is important for cancer metabolism and tumour growth. *Nature* 2008, 452(7184):230–233. 10.1038/nature06734 18337823

[31] Uphoff CS, Schoenebeck CW: Quantifying inter-population variability in yellow perch sexual size dimorphism. *Journal of Freshwater Ecology* 2012, 27(4):507–516.

[32] Schoenebeck CW, Brown ML: Does Anaerobic Activity Differ Seasonally or between Sexes in Yellow Perch Populations? *Transactions of the American Fisheries Society* 2012, 141(1):199–203.

[33] Henderson BA, Collins N, Morgan GE, Vaillancourt A: Sexual size dimorphism of walleye (*Stizostedion vitreum vitreum*). *Canadian Journal of Fisheries and Aquatic Sciences* 2003, 60:1345–1352.

[34] Doll JC, Lauer TE: Bayesian Estimation of Age and Length at 50% Maturity. *Transactions of the American Fisheries Society* 2013, 142(4):1012–1024.

[35] Ekblom R, Galindo J: Applications of next generation sequencing in molecular ecology of non-model organisms. *Heredity* 2011, 107(1):1–15. 10.1038/hdy.2010.152 21139633 PMC3186121

[36] Eujayl I, Sorrells ME, Baum M, Wolters P, Powell W: Isolation of EST-derived microsatellite markers for genotyping the A and B genomes of wheat. *TAG Theoretical and applied genetics Theoretische und angewandte Genetik* 2002, 104:399–407. 10.1007/s001220100738 12582712

[37] Buermans HP, den Dunnen JT: Next generation sequencing technology: Advances and applications. *Biochimica et biophysica acta* 2014. 10.1016/j.bbadis.2014.06.015 24995601

[38] Hawkins RD, Hon GC, Ren B: Next-generation genomics: an integrative approach. *Nature reviews Genetics* 2010, 11(7):476–486. 10.1038/nrg2795 20531367 PMC3321268

[39] Xiao S, Han Z, Wang P, Han F, Liu Y, et al: Functional Marker Detection and Analysis on a Comprehensive Transcriptome of Large Yellow Croaker by Next Generation Sequencing. PloS one 2015, 10: e124432. 10.1371/journal.pone.0124432 PMC4409302 25909910

[40] Wu C, Zhang D, Kan M, Lv Z, Zhu A, et al: The draft genome of the large yellow croaker reveals well-developed innate immunity. Nat Commun 2014, 5: 5227 10.1038/ncomms6227 25407894 PMC4263168

[41] Ma K, Qiu G, Feng J, Li J: Transcriptome analysis of the oriental river prawn, Macrobrachium nipponense using 454 pyrosequencing for discovery of genes and markers. *PloS one* 2012, 7(6):e39727 10.1371/journal.pone.0039727 22745820 PMC3380025

[42] Hood L, Hartwell L, Fischer J, Aquadro CC, Goldberg M: Genetics: From Genes to Genomes (5 Edition). New York: McGraw-Hill Education 2014, 816pg.

[43] Assis R, Zhou Q, Bachtrog D: Sex-biased transcriptome evolution in Drosophila. *Genome biology and evolution* 2012, 4(11):1189–1200. 10.1093/gbe/evs093 23097318 PMC3514954

[44] Conesa A, Götz S, García-Gómez JM, Terol J, Talón M, Robles M: Blast2GO: a universal tool for annotation, visualization and analysis in functional genomics research. *Bioinformatics* 2005, 21(18):3674–3676. 10.1093/bioinformatics/bti610 16081474

[45] Moriya Y, Itoh M, Okuda S, Yoshizawa A, Kanehisa M: KAAS: an automatic genome annotation and pathway reconstruction server. *Nucleic Acids Res* 2007, 35:W182–W185 10.1093/nar/gkm321 17526522 PMC1933193

[46] Li H: Aligning sequence reads, clone sequences and assembly contigs with BWA-MEM. arXiv: 1303.3997 [q-bio.GN], 2013.

[47] McKenna A, Hanna M, Banks E, Sivachenko A, Cibulskis K, et al: The Genome Analysis Toolkit: a MapReduce framework for analyzing next-generation DNA sequencing data. Genome research 2010, 20: 1297–1303. 10.1101/gr.107524.110 20644199 PMC2928508

[48] Thiel T, Michalek W, Varshney R, Graner A: Exploiting EST databases for the development and characterization of gene-derived SSR-markers in barley (Hordeum vulgare L.). *Theor Appl Genet* 2003, 106(3):411–422. 10.1007/s00122-002-1031-0 12589540

[49] Rozen S, Skaletsky H: Primer3 on the WWW for general users and for biologist programmers. *Methods Mol Bol* 2000, 132(3):365–386. 10.1385/1-59259-192-2:365 10547847

