# Supplementary material for: De novo transcriptome sequencing and analysis of male, pseudo-male and female yellow perch, Perca flavescens
Source: PLoS One. 2017 Feb 3;12(2):e0171187. doi: 10.1371/journal.pone.0171187 (PMC5291366; doi:10.1371/journal.pone.0171187)
Supplement: S1 Table — (DOCX) [file pone.0171187.s001.docx]

**S1 Table. Number of raw reads, gene counts and specifically expressed genes of yellow perch transcriptome sequencing samples.**

| **Sample** | **Number of raw reads** | **Number of reads after trimmed(paired+orphans)** | **gene count** | **Specifically expressed genes** |
| --- | --- | --- | --- | --- |
| FG | 50,038,026 | 49,955,444 | 99,279 | 17 |
| FM | 90,988,070 | 87,034,595 | 87,814 | 16 |
| MG | 114,305,962 | 108,656,555 | 207,349 | 6,476 |
| MM | 114,324,326 | 109,713,455 | 129,898 | 73 |
| PG | 98,627,630 | 93,765,841 | 196,391 | 665 |
| PM | 103,790,110 | 99,573,786 | 95,985 | 2 |
| Total | 572,074,124 | 548,699,676 | 211,976 | / |
