# Supplementary material for: De novo transcriptome sequencing and analysis of male, pseudo-male and female yellow perch, Perca flavescens
Source: PLoS One. 2017 Feb 3;12(2):e0171187. doi: 10.1371/journal.pone.0171187 (PMC5291366; doi:10.1371/journal.pone.0171187)
Supplement: S2 Table — (DOCX) [file pone.0171187.s002.docx]

**S2 Table. Mapping results of six tissue samples.**

| **Reference sequence** |  |  |
| --- | --- | --- |
|  | total reads | 147,454,266 |
|  | total contigs | 211,976 |
| **FG** |  |  |
|  | total reads(paired) | 49,947,140 |
|  | reads mapped in pairs | 33,210,258 |
|  | reads mapped in broken pairs | 9,640,140 |
|  | reads not mapped | 7,096,742 |
| **FM** |  |  |
|  | total reads | 84,358,602 |
|  | reads mapped in pairs | 52,923,804 |
|  | reads mapped in broken pairs | 15,857,426 |
|  | reads not mapped | 15,577,372 |
| **MG** |  |  |
|  | total reads | 105,146,864 |
|  | reads mapped in pairs | 80,855,044 |
|  | reads mapped in broken pairs | 10,672,251 |
|  | reads not mapped | 13,619,569 |
| **MM** |  |  |
|  | total reads | 106,867,740 |
|  | reads mapped in pairs | 69,307,454 |
|  | reads mapped in broken pairs | 20,301,952 |
|  | reads not mapped | 17,258,334 |
| **PG** |  |  |
|  | total reads | 90,734,840 |
|  | reads mapped in pairs | 62,449,576 |
|  | reads mapped in broken pairs | 17,032,590 |
|  | reads not mapped | 11,252,674 |
| **PM** |  |  |
|  | total reads | 96,877,950 |
|  | reads mapped in pairs | 60,330,912 |
|  | reads mapped in broken pairs | 18,769,211 |
|  | reads not mapped | 17,777,827 |
