# Supplementary material for: De novo transcriptome sequencing and analysis of male, pseudo-male and female yellow perch, Perca flavescens
Source: PLoS One. 2017 Feb 3;12(2):e0171187. doi: 10.1371/journal.pone.0171187 (PMC5291366; doi:10.1371/journal.pone.0171187)
Supplement: S3 Table — (DOCX) [file pone.0171187.s003.docx]

**S3 Table. Overview of sex-biased genes/ORFs identification.**

| **Item** | **Number** |
| --- | --- |
| NF-biased contigs | 3 |
| Annotated NF-biased genes | 0 |
| NM-biased contigs | 1440 |
| Annotated NM-biased genes | 55 |
| PM-biased contigs | 93 |
| Annotated PM-biased genes | 4 |
| specifically expressed ORF for NF | 0 |
| specifically expressed ORF for NM | 19 |
| specifically expressed ORF for PM | 6 |
