# Supplementary material for: De novo transcriptome sequencing and analysis of male, pseudo-male and female yellow perch, Perca flavescens
Source: PLoS One. 2017 Feb 3;12(2):e0171187. doi: 10.1371/journal.pone.0171187 (PMC5291366; doi:10.1371/journal.pone.0171187)
Supplement: S6 Table — (DOCX) [file pone.0171187.s006.docx]

**S6 Table. The Enzymes and related pathways involved in female, pseudo-male and male of yellow perch.**

| **Sex** | **Pathways** | **Enzyme code** | **Enzyme name** | **Number of hits** |
| --- | --- | --- | --- | --- |
| NF | none | none | none | 0 |
| PM | none | none | none | 0 |
| NM |  |  |  |  |
|  | Neuroactive ligand-receptor interaction | |  | 4 |
|  |  | EC:3.4.21.4 | trypsin |  |
|  | Phototransduction |  |  | 2 |
|  |  | EC:2.7.11.14 | rhodopsin kinase |  |
|  |  | EC:4.6.1.2 | guanylate cyclase 2D/E/F |  |
|  | Influenza A |  |  | 1 |
|  |  | EC:3.4.21.4 | trypsin |  |
|  | Protein digestion and absorption | |  | 1 |
|  |  | EC:3.4.21.4 | trypsin |  |
|  | Chemokine signaling pathway | |  | 1 |
|  |  | EC:2.7.11.14 | rhodopsin kinase |  |
|  | Purine metabolism |  |  | 1 |
|  |  | EC:4.6.1.2 | guanylate cyclase 2D/E/F |  |
|  | Pancreatic secretion |  |  | 1 |
|  |  | EC:3.4.21.4 | trypsin |  |
|  | Endocytosis |  |  | 1 |
|  |  | EC:2.7.11.14 | rhodopsin kinase |  |
