# Supplementary figures and images for: De novo transcriptome sequencing and analysis of male, pseudo-male and female yellow perch, Perca flavescens
Source: PLoS One. 2017 Feb 3;12(2):e0171187. doi: 10.1371/journal.pone.0171187 (PMC5291366; doi:10.1371/journal.pone.0171187)

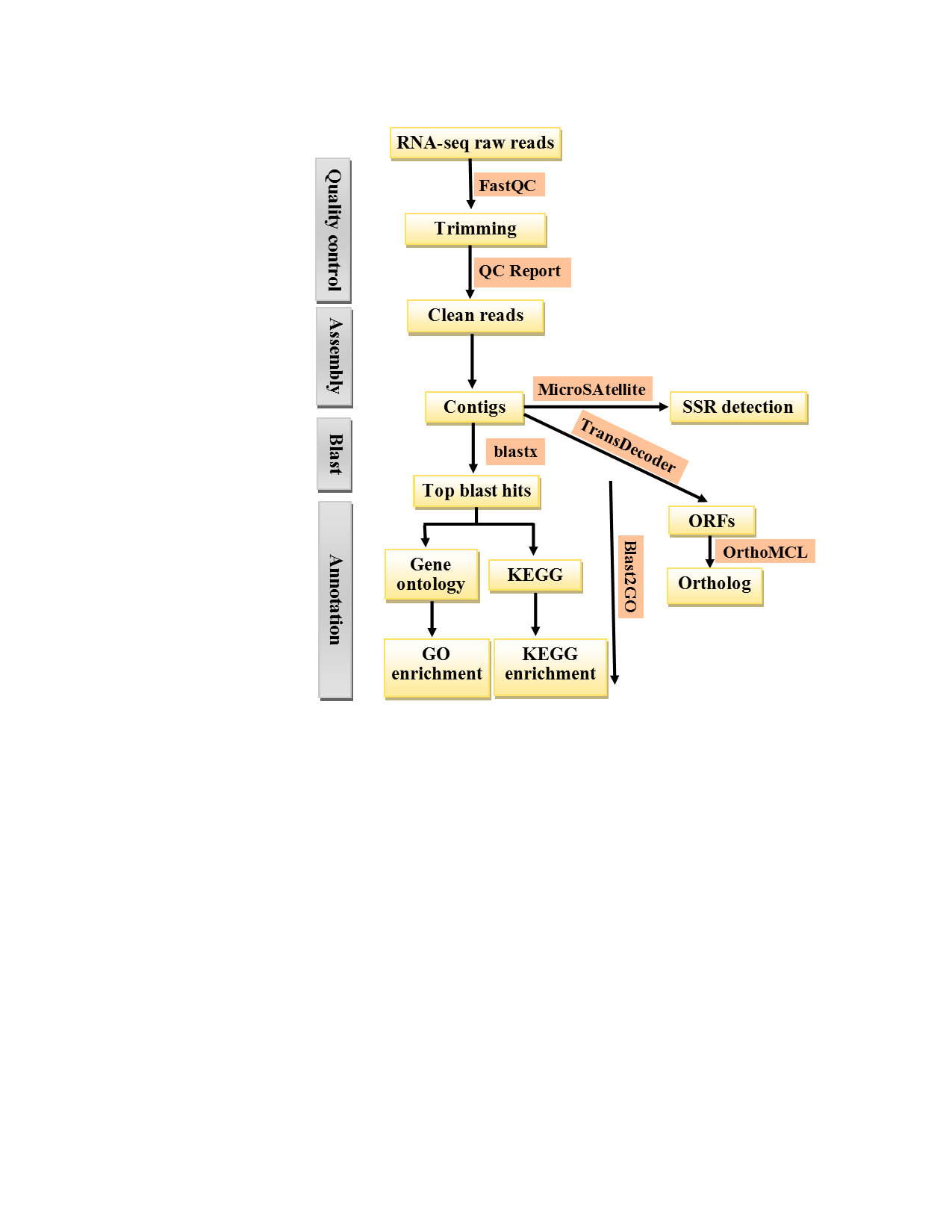


**S1 Fig. The workflow of transcriptome data analysis.**

Supplement: S1 Fig — (DOCX) [file pone.0171187.s008.docx]
