# Supplementary material for: De novo transcriptome sequencing and analysis of male, pseudo-male and female yellow perch, Perca flavescens
Source: PLoS One. 2017 Feb 3;12(2):e0171187. doi: 10.1371/journal.pone.0171187 (PMC5291366; doi:10.1371/journal.pone.0171187)
Supplement: S2 Fig — (DOCX) [file pone.0171187.s009.docx]

**The ORFs specifically found in NF (0):**

None

**The ORFs specifically found in NM (19):**

1. 5prime_partial len:138 (+) MG_S60_L005_R1_001_(paired)_trimmed_(orphans)_contig_103759:1-414(+)

CAGCAGCTGGAGCAGCAGCAGCAGCGGCGGCTCCTGTTTTCAGTGAAATCTAAGGGAGAGAGAGAGACAGGCAGCAAGAATTTTGTTACCAAGTCCAATATCCAAATGTCAGTTTTAGCCAAACTATATGTCTGTAAATTATATATATTAATGTCAGCTCTGCCTCCTCTTCCTCGTCTTTCTCCTTCTCTCTCTCCTTCTCCTCCACTTCCTTCTCCTTTTCCTCCTACTCTTCCTCCTCCTCTGCTTCCTTCTTCTCCTCCTCCTCCGGCACCTCCATGGGCATTAATATTCATCTTGGATGGATGGGTGATCTGTGGTTGTTCAAGTACAACGTTCATGTCTTCTGGGACTTTGGGCAACCTGTCACAGAAGGATTCAAACAGTTGTTCTTCTGTGTACTCACTAGTGTGA

2. complete len:118 (+) MG_S60_L005_R1_001_(paired)_trimmed_(orphans)_contig_118062:75-428(+)

ATGCCTGACATTACTTCTGTCCATTTTCCAAAGCATGCTTTAGTGCAGTGTTCCTCAACCTCTTTGGCTTGTGACTCAATTTGTTTCGTATCTCATTGCCCCCTTATCACAGGGGATAGACCTAGTGTGGCGATGGGTTGTGAACAATCATTTGGACAACCTAGTGTATTAGGAATGTGTTTTTCTGTCTTATCTGCATTTCAACACTCTTCTGATTTCCATTTTAGTGTTAGACCAAGGCTATGTATTTTGGGGCAGTGTAGGTGTTATGGTTTTAAGGTTCTAACTAGAGCCACAAACCACCTGGAATCAAAAGTTGCAATCAGTTGTCCAGTTTATGTATCCAGAGAATGA

3. 3prime_partial len:165 (+) MG_S60_L005_R1_001_(paired)_trimmed_(orphans)_contig_124896:89-580(+)

ATGTCTCTCTTCAACTCCAACTTTTGTCCCCTCCATGCCTCTCCTCCTTTTCAGGCCCCAATGTGTATAAACATGTCAGTCAACTCCTCTTCCTTTGCTGATCCCACTATTCTTCCTCCCAACGTTTCCTCCAACTCCTCCATCCATTCATGCTTCAGTTCTGGAATGACCATCTTCATATTCTCTACTTACTCCATCACCTCCATCCTCTCCCTTCCTGTCCTCATCTTCGTCCTCTACCTGGGTTACCAAGAGTGGAGGAAACAGCGCTCCTCTTCCACGGCTAAAAGTCATTCTGACGTCTTCACCTATCACTCCATCGTCATGCAGCTGGCTGAATTCTTGGGGTTCACTTCTTACATTTGTGGCAACTACATCAAGTGCTCAGTGATGATGAATGTAGCGTCAAATGTCTTGGCCTTCACCACCATCGGACAGGCTCTGTTTCAAGTCCTGAACTGTGTGGAGCACTACCTGGCTGTTGTTCACCCC

4. 5prime_partial len:126 (+) MG_S60_L005_R1_001_(paired)_trimmed_(orphans)_contig_141082:1-378(+)

TTGCATGTCTGTTCTCATCTCTCCATCTCCTTCATCCCTCACATACTGCTGCTGTCATCACTTCTTTATCACCTCTGCCCTTCGCTGCTCCTTCCTCTCCATCTATCCTCTCCTTCACTTCTCTCTCTCTATCTCCCCTCTCCTTCCCTTCTCTCTCTCTCTCACTCTCTCTCTCTCAAGGGATTGGTCACAGGTTCCGATTCATCTGATCGTTACGCCATCAGGCCCTGGGGCAATCTTACTGCAGCATGTATGCGTTTGTGTGTTTCAGCTCCAATTAGTGAAACTACATTTATGTATTTATGTGTGGATTTGTGTTTAAATGTATCTGGTAGCATTTGTGTGTGCACTGGTGTGTGTAGCCATGAAGGTGTTTAA

5. 5prime_partial len:102 (+) MG_S60_L005_R1_001_(paired)_trimmed_(orphans)_contig_146583:3-308(+)

ACAGGGTCAGAAAAAGCCATTTCAACAGGGCATATACCACATGGTTACATTGAGCAGTTCTTGCGCTCAAGACTTCTGTTCTCAGTTGTTTCTTACAGCATGTACTCAAATGGGCAAGTCACTGTTACTGGCAACACAGTCAGTATTTCCTCTTTTGATAGATATACTGTGGGAAGATGTTATTGCTATGTGGCCTTAGGAGGAAGTAGTTCTTACACACCATTTCAGAGAGACAAAGCAGCAGCTTTCACACAGTTTATTGATAAGTGGACTATCATTTTGTTAATGCCTGCTGGTTACCTGTAG

6. internal len:132 (+) MG_S60_L005_R1_001_(paired)_trimmed_(orphans)_contig_148651:1-393(+)

CGACAGCTGCTTCACCACGTAGTAGTCCATGAGGAAGGGGCAGCACACGGCCGCCACCAGCACGTCGGACACCGCCAGGTTGGCGATCAGCAGGTTGGTCAGGTTGCGCAGCTGCTTGTAGCGAGCGAGGCTGGCGATGAACAGACAGTTTCCCACCCCGCAGACCAACATGATGACCACCAGGACCACAGCGATGACAATGGTGGCCGCGAAGAAGGCCCGGCCCTGCGTGGTGTCAGGGATCTCGTCCACGGGCTCGTAGTCCAGACCGTCACTGGCGAGGAAGTAGTCTGGCAGAGCCGAGCTGCTGTTGGTCGGGGCGTCCATTCTGGGTCAGGTCAACATGTTCAGGAGTCTGAAGCTGATCCTTCACGCGCGCTGATCCACCGACTG

7. 5prime_partial len:108 (+) MG_S60_L005_R1_001_(paired)_trimmed_(orphans)_contig_157238:2-325(+)

ATTAGTTACATTTCAGCAACTGCCGACAACACCACAACACCACAAAATCCATTTTTGCCATTATCCCCACAGCCTCAACATAACTTTGTAAGCCTTCCCGCTCAGGGCCGACCAAAACTGGTCCAACTTAGATGGCCTCGACTGTGTGGAGATGGAGGTCCTGCATATGCCCCCAATAGAGCCAATGGTAACGGCACATTTTCACCCTCGAGTGTCAACAGTGTGGGGCTTCGGGACGACACACAGTCAACGGATGAGGCGCTGGAATTCAGCCTTCTGCCACAATACGGGGGGGGTCTATCCTGGTCGTGCCACATCGGTTAG

8.internal len:109 (+) MG_S60_L005_R1_001_(paired)_trimmed_(orphans)_contig_160120:1-324(+)

AGCAACAACCGCTTCACCATAGACATCAGGGATACAGGTGACATCTTGTTGAAGATCTGTAATCTGATGCCTCAGGACACAGGGATCTACACCTGCGTTGCCATTAACGACCACGGCTCTGCGTCCTCCTCCGCCTCCATTAAAGTGCAAGGTATCCCAGCAGCACCTGGGAGACCGGTGGCCCAGGAGGCCAGCAGCACGGCAGTGATGATCCACTGGCCGCCTCCGGCTTCACCAGCTCACTGTGCTGCCAGCAGCTACACCGTGGAGTACAGACAGGAAGACTCGTTGCTATGGCAGCAGGTGGCCAGCAGCAGAGAGGAG

9. 5prime_partial len:112 (+) MG_S60_L005_R1_001_(paired)_trimmed_(orphans)_contig_166387:2-337(+)

TTTTTTAAGTTTTATTTTCTTTCGACCTTTGGTTTAAAGTTTGCCTTTAAACCAAACGAAGACAATCGCATTTCTATTGTCAAATTAATGCACTTCTTTCATCCTATATTAGCTTTAAATGCTGATTTTCATGATACATTAACAGGACTAATACATTTATTTAGTGACAATATTTCCCAGGTAAATATATTTAATCCACATTGTTGGCTCTTTTTGGCTCAAATTGGGAACCATCCACTTAAATATGGATACACCTGTCATTACAATCAGTGCAATTATAGTGCAGGAGTGACAGAAGACATTCCGGTTCATCACTCTCCGTTGGATTTTAGCTAG

10. internal len:106 (+) MG_S60_L005_R1_001_(paired)_trimmed_(orphans)_contig_172579:3-317(+)

GGGTTCCTGTTAACGCTCCCCTCCTCTCATCCTGAATATGTTTGGCCTGTGAGGTTCCTAATGAAACAGACCCACAAAGACGAGAAAGGCTGTGTTTGTTCATGCATGCACAATGACTCAGAGCGCAGGTCGTCAGCATACATTCAGCCACTTAATTCAGCCACTAATGTTGAGATTTTCTTGCGTAATGGCTGCCATTTCCCCATGTTAAAAAGGAAGAGACTCTTCCTGATCAACCTCCCATTTCATTGTGGAGTAACACAGGAGCAGCACATTTGCTGCAATACAAATTTAATAGATCCATTTCATGCATGC

11. type:complete len:127 (+) MG_S60_L005_R1_001_(paired)_trimmed_(orphans)_contig_17478:896-1276(+)

ATGTACTCATTCTTCATAGCTGTGGCCTCACAATCTAACACGATAACATTTCAAAAACTGTTACACAACCACATTGATTTTCATTACATTCTCCCACAGAAATATCAAGAGTATACCACTTCACGGTCACCAGCTCTCATCTCAATAAACAGCTGTGGGATATCAGAACCCATTGTTTTCTATGCAAGGCCACCCACTGGCAGTGTGACAGTGCAACACCTGAAACATCCTCCTGGATCCATTCTTCATAGATTTACAATCCATGTATCATTTTGTCATCCTGATTGTCCATATTGTAATTTTACTATGTTGGTCTATTCTGTACAAATTACATCGAATGTCTACCTGGGAGAGGGATCTCTCATCAGTTGTTCTTCCTGA

12. internal len:115 (+) MG_S60_L005_R1_001_(paired)_trimmed_(orphans)_contig_177559:1-342(+)

ACAGACCCCTATGATGTAGGATGTGTCCCCGCCCACCAGATCACAGTCAAATTGAGACCACAACAATGGCCCTGCTGGCGTCCACAATACAGACTCAAACAGGAACAGGTGAAAGGGATTGAAGACACCATACAAGGACTACTCACAGCAGGTGTTTTAAGACCAACCAAATCCCCATGGAACACACCCATACTGCCAGTCCCAAAAGCAGAAAATAAAGGTTGGAGAATGGTACATGACCTACGACAAATCAATCAAGCTACCACTACTGAAAACATTCCCGTTCCAGACCCCTATGTTGCCTTACAGAATCTGAATCCCACCCACACACATTTCACTGTC

13.internal len:135 (+) MG_S60_L005_R1_001_(paired)_trimmed_(orphans)_contig_179043:2-403(+)

TGCCCCGTTGCAGCCCTGCTCGTATTTGAGAAGCCACAGTTGGAGTTCAGGGGAACGTACTATGTGCCGGCTAAAGGGGACCCTGTCGAAATGGGCTATGGCGCTATGGAACACGAACTGGGTACACGTCTCTGCGATGCGGAATTGGACGTCAACCATCTCATACTGAAAGCCAGCTTGCCTGTCATGATCGCATTGTTCTCAACAGCACTGACCGACAAATTCGCTCGCCAATACGAGACGATCGCCAAGAGTCAGGTGTTGAACAACTCGTGGAAAGACCCACGTTTCTCCTGGCCGACCAAGATATTCGAGCTAGCCGAGGCGATACAATTCGCTGGCAAGCCCACGCTGCATTGCTCCAGAGAGATGTCCTCGGTTGTGAAGGAGGCAGTGTTGAAG

14. complete len:110 (+) MG_S60_L005_R1_001_(paired)_trimmed_(orphans)_contig_179349:38-367(+)

ATGGAGGTGGCTGAGGGGTTAACAACTCATTACCTCACCCTGCTGCGCTCCACGGTGAGGTACAGTACGGGACTGTGGTGGCGGTGTTTGTGTCTTCTTCGTAACCCACAGCGCACCTTTAGGCTGTTACTCAACAAAGGGGAGCTCAGTGCAGTGACATCACCACAGAGACACTACGCCTGTGTGAGGGAGGCAAGTGGGAGACAAGGGCGTGGAGGATGGAGACTGTGGAGGCTGTGGACTGTGTGGGAGTCCCATTACTCCCCTCCAGCTCTTCTCTACCTGTCACATAAGCACTTTGAGGACTCAGCAGTAAAGCCACAGTCTTGA

15. internal len:107 (+) MG_S60_L005_R1_001_(paired)_trimmed_(orphans)_contig_185356:3-320(+)

CGCTTGAAGAAGAAAGGTGAGGAGAGAATGGCCCTTATGGAGAAGAAGATCCTGGAGCAGGTCAACAGTCTCTTCATTGTAAACCTGGCTTACGCTTATGACAACAAGACCCACCTGTGCCTGGTCATGGACCTTATGAATGGAGGAGACCTTAGATTCCACATCTACGAGCTCGGAGAGCGGGGTATCCGTATGGAGCGTGTTGTTTACTACATGGCCCAGATAATCACTGGGCTACTCCACCTGCACTCTATGGACATTGTGTACATGGATATGAAGCCTGAGAACGTGCTTCTGGATGCTAAAGGACAGTGTCGG

16. 5prime_partial len:147 (+) MG_S60_L005_R1_001_(paired)_trimmed_(orphans)_contig_53441:2-442(+)

CCAACTTTGTCAGGAACATCACTGCCAGCCCACGTCTCCACGTTAGCGATCAGACTCAAAAGCTGGTGTTTCTCAGCTTCACTGTGTTCAGAGGCGGACATGTCATCGCACAAGTTACTGGACATCTTCAACTGGCGAATCAAATATCTCAAGAGGTTACTGCCAGCAATGAGGTCATCCCCCTGGTTTTCAACGACGAGTGTGGGGACGGCCACACGACAGCCGTACACCTCCATCTCCAGCCCACACACTCCTAGAGGCTTCGTCCTTGACCCACCGCACCCAATCAAAATTACGTCTGAAGGTGCCAATGAAGGATTCTTTAACAGTCTCTTCTCCAACAGTCTGTTCCACATCTTGGAGCTTAAAGAACAAGCCATCGATCCGCTGTCCAGCATTGCCCTCACTTCAACGTTGCCATCAAGTAATACTTTTTCATAG

17. internal len:344 (+) MG_S60_L005_R1_001_(paired)_trimmed_(orphans)_contig_54022:2-1030(+)

TTGAACTACCAGCCCATGTGTACGGGTATGTTGTTTGAGAAAATGTACTACATGTTGAACAAAATGTCATCCTATGAATTGGCAAACCGCATGTACGAGACCGTGGCAAAACCGCTACGCGGCCAGCAAGATCTGGGACCTAAAGCGGCCATTGAGTTTGTGCAATCCAATCCATTGTGGCTGGCGGAACACGACACGGTCATCATACCCACTAAAATCACCGTCCCCATGGGCATGTCCGACAGGTACACATTCAACGAGCTCACTTGGTACACGGGTAGTTTGGGGAACAAGTTATTGCTCGCAGCAAACAAGAAGCATATTGAGAAATACAACTCGCAATACGACATTACCAGAGGCCTCAATTGGACCAAAAGGGACTCATTGAGTGGCTTCTTGGTGGACACGCTGGACATCTCGCTGGTCGTGGAAAGCTTCCACTGCGGCCTGTTTGACACATTGCAAGCCCACGTCACCCTTAGTGACAAACGGCGCAAAAGCTTGATGGGGCTATCTGACATGCTTAGTGTCAACTGTGTGTGGAAACCAAAGCTGAGCACGGCCTGCAATTGCAGCCGATCACTACTGGACTACATCCACCCCGAAGGCCTGTGCTCACCCACGTGTCTCTCGACACCGTTCGCTCACCTGTCAAAAGGAGTCGAGGGCAGCAATAATGACTGGACTCTGGAAATCACACGCACCCAACCCGACACCTCAATAAGGAGCCAGGGTAGGGTTCTGTACGGTTCGGTCCCCGCCACGACTGAAAGCTTCTGTCTAGTCAAGATGGACATTTTCACCCGCTCGCCTGGACCGAAACGAGACGCGCCATCTGCGCCTCTGCATGTGCTGCGATGTGACGTGGTATTGGTCGAGTTTGACATTGTGATACAAGACCGCACACCGGCTCTCGGTGAACAGCACTACTTGCACATAGCCGTACGATCCTTGTCAAAGGATGAGGACAACGCAAACATGCACGAGCTGCACGTGCCACTTGCGGCCGTGGACACCTTGAAAACTGTC

18. 5prime_partial len:170 (+) MG_S60_L005_R1_001_(paired)_trimmed_(orphans)_contig_78111:2-511(+)

GACGACGACGAGCTGTCACTGCTGCAATTGGAGTCCTGGCACGGCGTCCCGGGAAGGCTCGGCCTGCTGACTACTGGGTGGACGCGTTCCCGGCCGGAGGTCTGCGCCCTCTCCCGCGGGCCCAGGCCGTGCTCCTTCTTGCAGCCGAAGTCCGGCCGCAAAATGTTGTCGATGAAAAAGTTGGTGGTTCTGTGGGCCTGCTGGGCGACCTGCAGAGGCAGTAGGGGCGGCGAGGGCAAACTGGGAGAGGGGGAGACGCTCTCTCCCTCGCTGCTATCCGTGCTGTTCAAGTCCCGCTCTTCCTCCATCGCTGCTTCTCTGAAGAACGTGGCTTCTCTTCTCTCAACCCACTTCCACTTTGCAGGCCTGTGTTTGTATCCAAAATATGACAACAGGAAAGCACTAAAACATAGTTATTTTCTCTCCGAAATATTGCTGGTTGGTGTAATTATTCGCAAACTGTGGACATCCTGCCTGCGTAAAGGCGCGCCGGCGCGTCATGCTCGGTGA

19. 3prime_partial len:110 (+) MG_S60_L005_R1_001_(paired)_trimmed_(orphans)_contig_98219:63-389(+)

ATGGACTTGAAAGCCTGTCGCTGTATATTGCTTTCAAATCTCAAATATACTCTTCTCAGTCTTGTGAACTCCCTTGCTGCCCTCTCCTCTTCTTCTCTTTGTCTTTCTCACTCTCTCACACTTACATACTTAATTGCAATAACTATCTCTTCCTCTTCTTCATCTTTTTTCTCCATCTCTTGTCTCTGTTTTCATCCTTATCCTCATGCTGTCTTGGTGTCTAACCCTCTTGTTTTGTCTCATTTCATACAACATCCTACATTTCTTTTTCCTCCTCTTCTTATATCCTTACATCCCCTCTATCTCCCCTTCTCTCTTTCACTTTCT

**The ORFs specifically found in PM (6):**

1. internal len:110 (+) MG_S60_L005_R1_001_(paired)_trimmed_(orphans)_contig_13061:1-327(+)

GAGGAGCACAAGATTCTGACTTCTCTTCAAGATGTGAAATCCAAACTGTTCTTTGTTGTTAACCGCAAGGAGGGGAATGTTACAGAGGATATGATGTCTGTCGAGAGAACACAGAAAGAATTGGACTTACCAAAGACCAGTATAAAAACCAAAGACCCAAGGGTAAACGTTGCAACGTTTTCAGATAAATTGTGTACAGCCATCAAGAAATCACTGACTGATGTAACAACCACAATGACAATTGAAAATATGCTTGAGAGAGCTGTTGAACTTGGTCTGTCTGTGGATGAACACACAAGTGATGACGAAAAGAAAGCAGCTCAGGAG

2. 3prime_partial len:131 (+) MG_S60_L005_R1_001_(paired)_trimmed_(orphans)_contig_20657:664-1053(+)

ATGTCTTTGGCTGGTTTGAGATCAGCTACGGTGAGCAAATCTGCCGGGTCACCCTTTGGATCATGTTTGTGTGTTTTGAGGTATTCTTCAATGGCCTTTCGTTCGTACACATTTCCGTATTTTGTTCTCACAGGTTCAACAAACATCTTGTTGGTGAGTGGACAGATTACATGTTGGGGTGCAAGCTGTTTCCCAGGATCCACCAATNNNNNNNNNNNNNNNNNNNNNNNNNNNNNNNNNNTCAGGGATGATGAGCAGTCCATATATAGCATTGAGGATATCTCTCATGGTGATTTGGGCATTGTAGTTCCGGTCAAAAATGTTGTGGCAGATGCGCCCAACACTGTTGATATTACAGTGGTAGATACGTGTGACAAAGCGAACAAGTGG

3. 5prime_partial len:101 (+) MG_S60_L005_R1_001_(paired)_trimmed_(orphans)_contig_208420:3-305(+)

GCACCTTGTCTGGATTCCGGGCAGTTGTTGCTAAATGACAAAGAAAACAGCTTTCATGTGGATGAAAGAGGAGTCTTCATCTTAGAAGGGGACGGGTCCGTAGTAGGCGACGTATGCTTTGACGATGCCTTCTGTTTCTGCCATCTCGTCACAGGCGTCGTTCAGCTCACACACCTCTCTCAGGCTTTCCAGCTGCAGAGGGCTGAGGTCTCCTGCTGCGGCAGCTCTTCTTCTCCTCAGGAGAACAGCAGCCAGGTCTCTCTTCATCACCACCTGAGCCGCCTCAGCACTGGCGGACTCTGA

4. 5prime_partial len:103 (+) MG_S60_L005_R1_001_(paired)_trimmed_(orphans)_contig_208647:2-310(+)

CAGGGTGTTGTAGAACTGCGGCCGGTTGATCCCTTTGTCCAGTTCATCTTTTTTGGGCACAGACCTGCCCAGGGTATGGCGCCCATCCATGACCCCCATGCTGACCATGTTAGCTTTGGCTCCCCGGGTGCAGCTCTGCTGGAAGCCAAAAGAGGGCATTGTACCGGTTATGGCGGTGGTGATAGGGGTGGAGGTCGGGCTGTTGCTGCCAGGACCCTGGAACTGGGCCAGGGCTGGGGGCATCCAGCAGCTGTCGGAGTGGCCCAGTATCAGACATTCCTGGGTACAGGTCTCCGAGGCCTCACCTAA

5. internal len:112 (+) MG_S60_L005_R1_001_(paired)_trimmed_(orphans)_contig_209107:1-333(+)

CACAGGTCTGGCCTTGATATGAATTCTTTCAGCGTCATTTGCTCCCTCCTTACCGCACCCCTTGTGTTAAGTACCTTCTTAATGTTCTTAATCACCTCCAACCTTGTCATCTCAGCTCTGTTGAACTCCCCCGCACACCAATTCTTCAGTATCTCGTCTTGCTCTTGCTTTGGTACCTTGTTTAGGTTGAACCCCCCTAGCATATTCAAATCTACCAGCGCCCTCCACCCACTTATCCTTGGCCCTCTCCTTTTCACCCAGTCTGACAATTGTTTTGCTCGCTCTGACCAGACCTTCTTACCCACTGTTTCTAATAAGCCGCACTGCAACATC

6. internal len:110 (+) MG_S60_L005_R1_001_(paired)_trimmed_(orphans)_contig_210181:1-327(+)

CTTCCCTCCATCTTTCAACTGCTGGCTTTCTTATTCTCATTCATACAGTCACTCACAGAGACTCAGTCTAACCCTATAATCTTCTTTCCTTCTCCACTTACCCTTCACCAGCACACATACACACTCAGCTTATGTGTTTGTGTGTCTGCCTTTGGTGTATATGTGTGTGTCAGACAATTCTGCTCACATACATGTTATGACGCGGTCATGAATCAGATGTTAGCGCAGCTTTTTGCCATAAGGACTCCTGCACGACATGCAGCCTCCTATGAAGCGCACAGGCGCAGCAATCTAACATGCTGCAAGCACAGCACTGCAGACTCTGAG

**S2 Fig. The ORFs specifically found in female, male and pseudo-male.**
